# Execution Velocity as a Task Constraint Modulates Motor Variability and Movement Complexity in Dynamic Resistance Exercise

**DOI:** 10.3390/s26196286

**Published:** 2026-10-04

**Authors:** Miguel López-Fernández, Fernando García-Aguilar, Carla Caballero, Francisco J. Moreno, Rafael Sabido

**Affiliations:** 1 Sport Research Centre, Department of Sport Science, Miguel Hernández University, 03202 Elche, Spain; fernando.garciaa@umh.es (F.G.-A.); ccaballero@umh.es (C.C.); fmoreno@umh.es (F.J.M.); rsabido@umh.es (R.S.); 2 Neurosciences Research Group, Alicante Institute for Health and Biomedical Research (ISABIAL), 03010 Alicante, Spain

**Keywords:** movement complexity, motor variability, execution velocity, resistance exercise, constraints, entropy, detrended fluctuation analysis

## Abstract

Execution velocity is a key constraint in resistance exercise, yet its influence on motor variability and movement complexity remains poorly understood. This study examined how different execution velocities modulate the magnitude and temporal structure of motor variability during a dynamic resistance task. Seventy-two resistance-trained participants performed parallel back squats at 50% of one-repetition maximum under three velocity conditions: preferred velocity, slow velocity, and maximal intended velocity. Acceleration signals were recorded using an inertial measurement unit placed at the lumbar region, while ground reaction force was obtained from a force platform. The magnitude of variability was quantified using standard deviation, whereas movement complexity was assessed through Fuzzy Entropy and Detrended Fluctuation Analysis. Repeated-measures ANOVAs revealed significant effects of execution velocity on standard deviation and Fuzzy Entropy in both measurement systems, with effect sizes of η^2^p = 0.937 and 0.738, respectively, for the IMU, and η^2^p = 0.882 and 0.712, respectively, for the force platform. Maximal velocity increased the magnitude of variability, whereas slow velocity produced higher complexity values, particularly for Fuzzy Entropy. Detrended Fluctuation Analysis showed a significant effect only in force-platform data (η^2^p = 0.182). These findings indicate that execution velocity modulates motor variability in distinct ways: faster executions amplify mechanical fluctuations, whereas slower executions increase the temporal complexity of the movement pattern. Therefore, velocity should be considered not only as a mechanical or physiological training variable, but also as a task constraint capable of shaping the organization of motor behavior during resistance exercise.

## 1. Introduction

In strength training, it is common to use constant constraints, such as load, range of motion, or speed of execution, during the same set. However, even if these constraints remain steady, variability persists across repeated movements [1,2]. Motor variability refers to variations in performance across repetitions or during continuous performance of the same task [3].

Motor variability can serve a functional role by allowing different movement solutions to achieve consistent task outcomes [4,5]. Its relationship with learning and adaptation has been well-studied [6,7], suggesting that motor variability is a form of adaptability that modulates motor output to enhance learning and adaptation. However, the functional meaning of variability depends on the characteristics and demands of the task [5,6]. Motor variability can be characterized through both its magnitude and its temporal structure. The magnitude of variability reflects the extent of fluctuations in the signal and can be quantified using statistical measures such as standard deviation [8]. However, measures of magnitude do not describe how these fluctuations are organized over time. In contrast, complexity refers to the temporal organization of variability, providing information about the regularity, predictability, and temporal correlations of the signal [9,10,11,12]. Therefore, the magnitude and complexity of variability represent complementary but distinct characteristics of motor behavior, and a greater amount of variability does not necessarily imply greater complexity. Importantly, greater complexity should not be interpreted as inherently favorable or as direct evidence of enhanced adaptability. In the present study, complexity is used descriptively to characterize the temporal organization of motor variability. Non-linear methods can be used to characterize this temporal structure, including Detrended Fluctuation Analysis (DFA) [9] and Fuzzy Entropy (FuzzyEn) [13,14]. These methods characterize different properties of the temporal structure of variability. FuzzyEn quantifies the regularity of a time series, with higher values reflecting a more irregular and less predictable temporal structure [13,14], whereas DFA characterizes the scaling properties and temporal correlations present within the signal [9,10,12]. Therefore, their combined use provides complementary information about the temporal organization of motor variability.

Within the training environment, variability causes muscle force production to exhibit constant fluctuations in muscle contraction and target force production [15,16,17]. Both the magnitude of variability and its complexity can be influenced by modifying or introducing different constraints within strength training [2,18,19]. The intensity of the load, the type of contraction, the velocity of execution, or the use of an external focus during exercise execution are examples of constraints that can be used to challenge the system and achieve a range of adaptations [2,18].

Some studies have shown that muscle force complexity decreases during fatiguing contractions [19,20]. Moreover, Moras et al. [18] found changes in movement complexity in a dynamic strength exercise when an external element, such as a rugby ball, was introduced. Additionally, increasing intensity has been demonstrated to be a constraint that leads to increased movement complexity in dynamic strength tasks [2].

One of the most studied training variables in recent years is velocity during resistance exercise [21,22]. In everyday tasks such as walking, gait speed has been shown to induce changes in movement complexity [23,24]. This suggests that modifying execution velocity can influence the temporal structure of movement variability. However, despite extensive research on the physiological and mechanical effects of velocity in resistance training [25,26,27], the impact of execution velocity on movement variability and motor behaviour has been hardly examined in muscular strength tasks.

As execution velocity may influence movement complexity, it is of particular interest to determine how different execution velocities modify the temporal organization of motor variability during resistance exercise. Therefore, this study aims to understand how the application of varying execution velocities in the back squat exercise influences both the magnitude and the complexity of motor variability. Based on previous evidence suggesting that higher movement speeds tend to constrain the system’s ability to make fine motor adjustments [23,24], we hypothesized that slower execution velocities would elicit greater movement complexity, whereas faster execution velocities would elicit lower complexity.

## 2. Materials and Methods

### 2.1. Participants

Seventy-two healthy participants (age = 25.7 ± 4.4 years; height = 169.2 ± 9.8 cm; body mass = 67.7 ± 11.2 kg; one-repetition maximum (1RM) in squat = 95.5 ± 28.7 kg; ratio 1RM/body mass = 1.4 ± 0.3) took part in the study (Table 1). No a priori sample size calculation was performed, as recruitment aimed to include the maximum feasible number of participants within the study period. A post hoc sensitivity analysis was subsequently conducted based on the final sample of 72 participants, showing that effects greater than ηp^2^ = 0.02 could be detected with a statistical power of at least 80%. All participants had at least one year of experience in resistance training in the squat exercise, and they reported no injuries during the six months before the study. Participants were instructed to maintain their normal lifestyle, including nutritional and hydration states. Caffeine intake was not allowed within the 3 h preceding measurements. Additionally, resistance training sessions were not permitted within the 72 h preceding the experimental sessions. Before participation, each subject provided written informed consent. The study was approved by the Research Ethics and Integrity Committee of Universidad Miguel Hernández de Elche (authorization code COIR DCD.CCS.01.23; file number 2023/33565) and conducted in accordance with the Declaration of Helsinki.

### 2.2. Procedure

Participants attended three test sessions at least 72 h apart. They were scheduled at the same time for each of the three sessions to avoid the influence of circadian rhythms and variability in the experimental data. In the first session, participants performed a 1RM parallel back squat test after familiarization with the warm-up protocol. A parallel squat was considered when the greater trochanter of the femur descended to the same level as the knee, leaving the femur parallel to the ground [28]. The 1RM was estimated by a linear position transducer (T-Force System, Ergotech, Murcia, Spain). A protocol was established for the 1RM test, requiring the load to be progressively increased until the propulsion speed was less than 0.5 m/s [21]. In sessions two and three, participants performed an experimental protocol consisting of three sets of four consecutive repetitions (executed continuously without pause) of the back bar squat to parallel. The appropriate depth of repetition was ensured by placing a small barbell at the individual’s height at which each participant was to descend, lightly touching it with the buttocks, and then performing the ascent. Participants performed three sets at 50% of 1RM. This intermediate load was selected to provide an appropriate balance between force and movement velocity, allowing high mechanical power production while enabling clear differentiation among the execution-velocity conditions, and was consistent with our previous squat-based motor variability protocol [2]. Each set was performed at a different velocity: preferred velocity (PV), slow velocity (SV), and maximal velocity (MV). PV was performed at a velocity at which participants felt most comfortable, at 50% of their 1RM. SV was calculated by measuring the average time taken for the four repetitions at the PV multiplied by two (double time). A beeping metronome was used to cue the participant when to begin the up and down phases of the squat to match their individual slow velocity. Before data collection, participants were familiarized with the individually prescribed metronome-guided tempo without external load, allowing them to practice synchronizing the descending and ascending phases with the auditory cues. MV was performed at each participant’s maximum intended velocity in both the up and down phases, under the premise of executing “As fast as possible”. The order of the velocity conditions was fixed (PV, MV, and SV) and was not randomized. PV was performed first because the individual SV condition was determined from the duration of the PV repetitions. MV was performed before SV to minimize potential carryover fatigue, as the slower condition involved a longer time under tension and was perceived as more demanding during pilot testing. A four-minute rest interval was provided between sets, consistent with our previous squat-based motor variability protocol [2]. Given the low volume and moderate load used in the present study, this interval was considered sufficient to allow adequate recovery and minimize fatigue-related carryover between conditions.

### 2.3. Data Collection and Analysis

Acceleration signals were recorded using inertial measurement units (IMU) manufactured by STT-System (San Sebastian, Spain). The IMU device was placed in the lumbar region at the level of the iliac crest between the L3 and L5 vertebrae. A force platform (Kistler, Winterthur, Switzerland, Model 9287BA) was used to measure motor variability from force reaction during squat. Both IMU and force-platform signals were recorded at a sampling frequency of 100 Hz, which has previously been shown to provide adequate reliability for the assessment of linear and non-linear motor variability during the squat exercise [29]. Synchronization between the IMU and force platform was performed using a common trigger generated by striking the force platform with an IMU, following the procedure previously described by García-Aguilar et al. [29]. IMU and force plate signal were analysed by standard deviation (SD) as a linear measure of variability. As a non-linear measure to examine the variability structure of the acceleration modulus time series, Detrended Fluctuation Analysis (DFA) Long-Term for fractal scaling, and Fuzzy Entropy (FuzzyEn) was used to analyse the regularity (predictability). The modulus is the result of the values recorded on the Z, X and Y axes. FuzzyEn was calculated according to Chen et al. [14] using the following parameters: m = 2, r = 0.2 × SD, and n = 2. This parameter configuration was selected based on previous applications in squat-based motor variability analyses, in which it showed adequate reliability [2,29]. DFA was computed following the method proposed by Peng et al. [30], using the same window range across all velocity conditions (4–100 data points, corresponding to a maximum window duration of 1 s at 100 Hz), consistent with our previous squat-based analyses [2,29].

In the context of this study, the term movement complexity refers to the temporal organization of motor variability, as captured by the non-linear measures. Specifically, greater complexity denotes a more irregular and less predictable signal over time, that is, lower temporal autocorrelation and higher entropy values. In this sense, higher FuzzyEn values indicate a richer, less repetitive temporal structure in the variability signal, whereas lower values reflect more regular, predictable fluctuations. For clarity, throughout the manuscript, the term complexity is used to denote the structure of variability, describing these non-linear temporal properties.

### 2.4. Statistical Analysis

The data obtained were analysed using JASP 0.17.1.0 (Jeffreys’s Amazing Statistics Program) [31]. The normality of the variables was assessed using the Kolmogorov–Smirnov test. Repeated-measures ANOVAs with one factor (3 × 1) were then performed to examine the effect of execution velocity on the variables SD, DFA and FuzzyEn. Sphericity was assessed using Mauchly’s test. Outlying observations attributable to signal acquisition problems were excluded from the corresponding analyses. A post hoc analysis was performed to analyse pairwise comparisons, and the Bonferroni correction was applied. The level of statistical significance was considered *p* < 0.05. Partial eta squared (η^2^p) was calculated as a measure of effect size and to provide a proportion of the total variance attributable to the factor. For descriptive interpretation, partial eta-squared values of approximately 0.01, 0.06, and 0.14 were considered indicative of small, moderate, and large effects, respectively, consistent with thresholds previously applied in motor variability research [32].

## 3. Results

**Figure 1:**
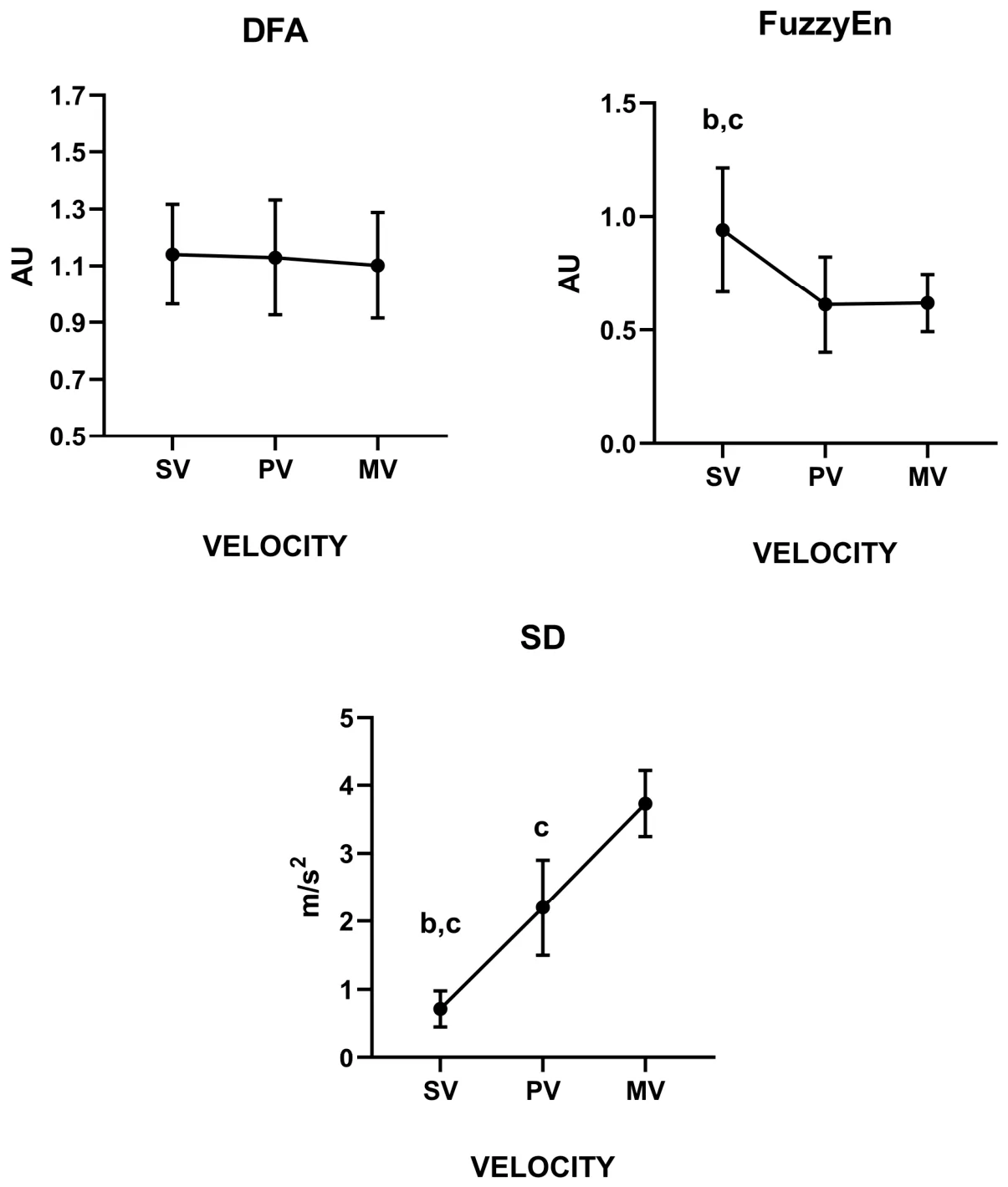
IMU-derived motor variability measures across execution-velocity conditions. Values are presented as mean ± SD. Letters indicate significant pairwise differences: b = difference with respect to PV; c = difference with respect to MV.

The ANOVA revealed significant differences between execution velocities on the IMU device for SD (F = 891.671; *p* < 0.001; η^2^p = 0.937) and FuzzyEn (F = 169.196; *p* < 0.001; η^2^p = 0.738), whereas no differences were observed for DFA (F = 0.960; *p* = 0.386; η^2^p = 0.016). Pairwise comparisons showed that for SD, all three velocities differed significantly from one another. The SV condition was primarily responsible for the observed effects in complexity-related measures, showing significantly higher FuzzyEn values compared to both PV and MV, while no significant differences were detected between PV and MV. This pattern indicates that SV plays a central role in driving the differences in complexity-related measures, while maximal velocity only amplifies variability in terms of SD (Figure 1).

**Figure 2:**
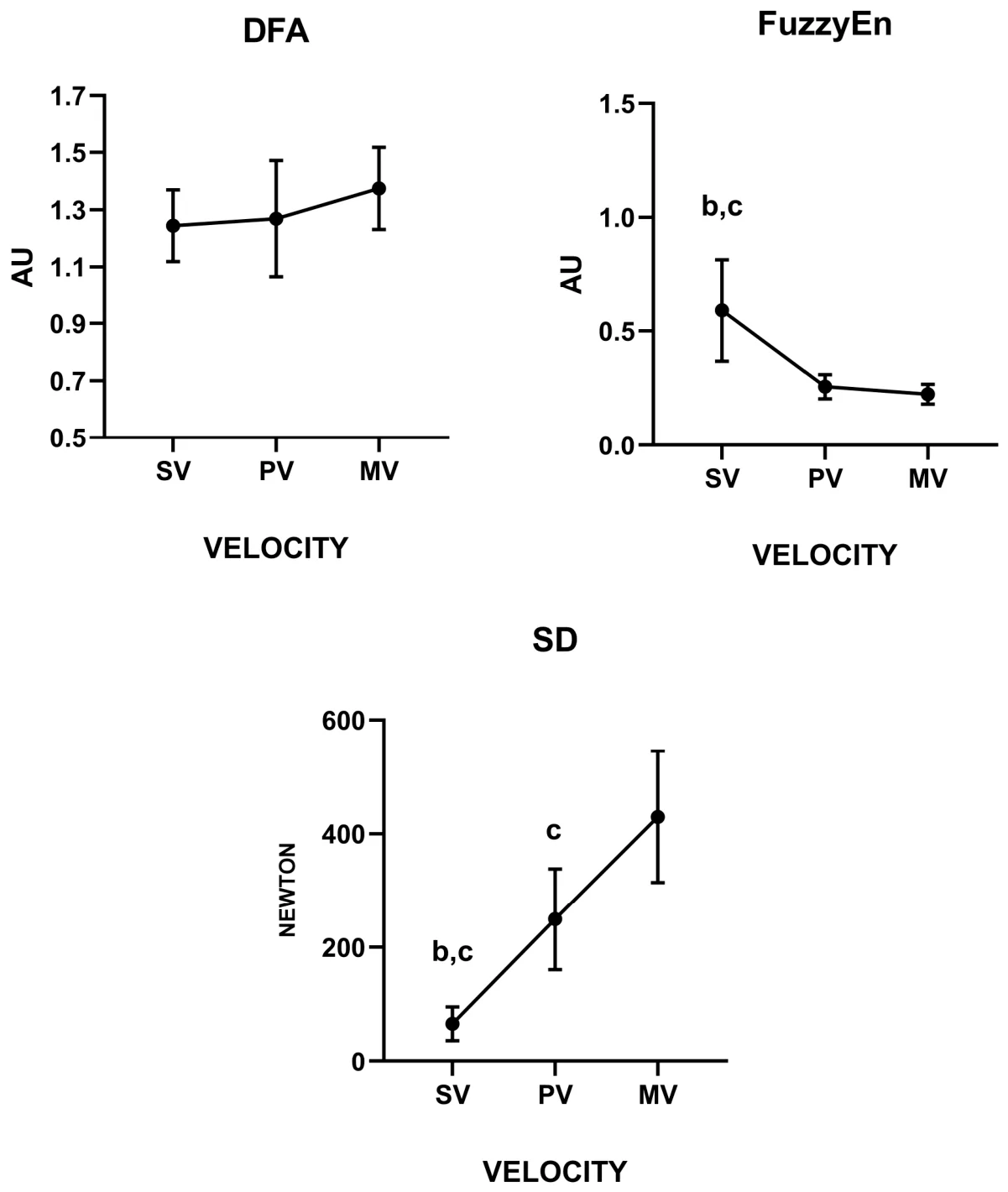
Force-platform-derived motor variability measures across execution-velocity conditions. Values are presented as mean ± SD. Letters indicate significant pairwise differences: b = difference with respect to PV; c = difference with respect to MV.

Force-platform data revealed significant differences were found between the different execution velocities for SD (F = 480.192; *p* < 0.001; η^2^p = 0.882), DFA (F = 14.025; *p* < 0.001; η^2^p = 0.182) and FuzzyEn (F = 156.118; *p* < 0.001; η^2^p = 0.712) measures. Pairwise comparison using DFA revealed significant differences between most execution velocities, except between SV and PV. Similarly, FuzzyEn showed significant differences among the velocity conditions, except between PV and MV. With SD, significant differences were observed across all execution velocities. Figure 2 shows the significant differences between each of the execution velocities on the force platform.

## 4. Discussion

The purpose of the study was to evaluate the influence of different execution velocities in the squat exercise on the amount and complexity of variability. The analysis of motor variability is of increasing interest due to its relationship with learning and adaptive capacity [33]. Studies indicate that constraints influence movement variability [34,35].

Overall, our results show that execution velocity affected different properties of motor variability in distinct ways. SD progressively increased with execution velocity, whereas FuzzyEn was higher during the slow-velocity condition and did not differ between preferred and maximal velocities. These different responses are consistent with the distinct information provided by these measures: SD quantifies the magnitude of fluctuations, whereas FuzzyEn characterizes their temporal regularity and predictability. Therefore, changes in the amount of variability do not necessarily imply equivalent changes in its temporal structure. Similarly, although both FuzzyEn and DFA characterize the temporal structure of variability, they describe different properties of this structure. FuzzyEn reflects the regularity and predictability of the signal, whereas DFA characterizes its scaling properties and temporal correlations. Accordingly, these measures may differ in their sensitivity to changes in execution velocity. Moreover, DFA showed significant differences between velocity conditions in the force-platform signal but not in the IMU signal. This difference should be considered in relation to the distinct mechanical signals captured by each measurement system, namely ground reaction force and lumbar acceleration. The increases in complexity of the signal observed in our study indicate that the temporal organization of the signal changed across velocity conditions. These changes may reflect different patterns of moment-to-moment adjustment to task demands [2,18].

### 4.1. Effects on the Amount of Variability

Regarding SD, our results suggest that performing squats at higher velocities is associated with greater movement variability, likely due to the higher velocity peaks achieved and the rapid transitions between movement phases. Conversely, at slower velocities, the amount of variability is lower, which could be explained by lower peak force production compared to higher velocities.

An opposite influence of movement speed on variability has been reported in trunk flexion–extension tasks: Asgari et al. [36] reported that slower velocities (20 cycles/min) were associated with greater variability compared to faster speeds. However, it is important to note that their study analyzed cycle-to-cycle variability in trunk and pelvic angular trajectories, recorded through a 3D motion capture system. Variability was quantified using the coefficient of variation and standard deviation of joint angles across repeated cycles, reflecting the consistency of movement patterns between repetitions rather than the moment-to-moment fluctuations within a single continuous execution. In contrast, the present study assessed within trial variability, that is, the fluctuations occurring during a single continuous execution of several repetitions under load. These two types of variability capture different aspects of motor control: inter-trial variability relates to consistency across repetitions, whereas intra-trial variability reflects the dynamical adjustments made moment to moment in order to cope with task demands.

From this perspective, the higher SD values at maximal velocity observed in our study suggest that faster squats amplify short-term fluctuations due to the increased mechanical demands of acceleration and deceleration phases. Meanwhile, slower velocities constrain those fluctuations, promoting more stable force output within each repetition.

This interpretation also helps to contextualize the findings of King and Hannan [37], who did not find differences in coordination variability between preferred and slow tempos when squats were performed without external load. The absence of load likely reduced the need for continuous motor adjustments, thereby minimizing intra-trial variability. In our study, the inclusion of an external load (50% 1RM) increased the mechanical demands of the task, increasing the dynamical complexity of the system and potentially magnifying variability responses to changes in velocity. Thus, the contrasting results across studies may reflect methodological differences in the task constraints and in how variability is quantified, particularly the presence of external load, the mechanical variable analyzed, and whether variability is assessed within or across repetitions.

### 4.2. Effects on Complexity

In general, the increase in execution velocity from SV to PV led to a decrease in movement complexity. However, from PV to MV, no significant changes in movement complexity were observed, except for DFA, which shows an even greater decrease in complexity in the force platform. This finding suggests that the prolonged time under tension and the greater temporal control associated with the lower execution velocity in SV may contribute to the higher complexity observed, potentially reflecting greater adaptive demands on the motor system.

This interpretation aligns conceptually, though not methodologically, with the results of Jordan et al. [23], who reported higher complexity at participants’ preferred walking velocity, with decreased complexity observed at both faster and slower speeds. However, it is important to note that in their study, variability was computed between gait cycles (i.e., inter-step variability), derived from the duration and length of each step. Therefore, their measure captured the adjustments occurring from one stride to the next, rather than the continuous fluctuations within a single execution.

In contrast, in the present study, complexity was assessed within the continuous execution of several loaded squat repetitions (intra-trial variability). This approach captures the moment-to-moment adjustments that the motor system performs to maintain control and stability under mechanical constraint. Performing the squat slowly under load requires sustained force production, precise temporal control, and continuous feedback integration, all of which may lead to a higher number of fine motor adjustments and, consequently, greater signal complexity [2].

Additionally, the use of a metronome to prescribe the slow-velocity condition introduced an external rhythmic cue during execution. Although participants were familiarized with the individually prescribed tempo before data collection, the influence of rhythmic cueing cannot be completely separated from that of slower execution velocity. Therefore, the higher complexity observed during SV may reflect the combined influence of reduced execution velocity and externally guided temporal regulation. This interpretation is consistent with Moras et al. [18], who reported increased movement complexity when an external element was introduced during a resistance task. Similarly, Tuyà Viñas et al. [38] found higher entropy values when squats were performed at slower tempos (40 bpm vs. 60 bpm), consistent with the idea that slower, externally constrained movements require a higher degree of motor coordination and control. Taken together, these findings suggest that the effect of execution velocity on movement complexity should be interpreted in relation to the specific task, the signal analyzed, and the temporal scale at which variability is quantified.

In our study, the slow-velocity condition combined mechanical load, prolonged time under tension, and an externally imposed rhythm. These constraints together may have increased the control demands of the task, potentially contributing to the greater movement complexity observed. This interpretation aligns with other findings showing that introducing novel or demanding constraints, such as higher loads [2] or task-specific decision-making components [39], elicits greater complexity in movement patterns.

### 4.3. Limitations

A potential limitation of this study is that the number of data points collected differed between velocity conditions due to the distinct durations of each repetition set. Although the number of repetitions was standardized across conditions, the sampling length inherently varied, with slow executions producing a larger number of samples than faster ones. This difference could have influenced the magnitude and structure of the recorded signals, potentially affecting the variability and complexity measures.

Truncating the time series to the same number of data points could have allowed for a more direct comparison between conditions but would also have implied analysing different numbers of repetitions across velocities. Conversely, time-normalizing all series to an identical data length would have altered the temporal resolution of the underlying adjustments made during movement, potentially leading to biased interpretations. Therefore, we opted for an ecologically valid protocol, maintaining a fixed number of repetitions in each condition, closely resembling real training practices. This approach preserves the natural temporal structure of the task, albeit at the cost of producing time series of unequal length.

A further limitation of this study concerns the use of a single relative load (50% 1RM) across all conditions. Although this intensity was selected to standardize the mechanical demand and facilitate the comparison between velocities, it may not fully represent the spectrum of neuromuscular responses elicited at higher or lower intensities. Motor variability and complexity could behave differently under greater or lesser load constraints, as the balance between mechanical output and control demand varies with intensity. Future studies should therefore examine whether similar patterns of variability are observed when different relative loads are combined with varying execution velocities.

Finally, no a priori power analysis was performed. Although the final sample showed adequate statistical sensitivity according to the post hoc analysis, prospectively determining the required sample size based on an expected effect size and desired statistical power would have strengthened the study design.

## 5. Conclusions

This study demonstrates that execution velocity during the squat exercise modulates motor variability in distinct ways: slower velocities increase movement complexity, reflecting greater demands on temporal regulation and movement control, whereas faster velocities amplify the magnitude of variability, indicating greater mechanical fluctuations. A decrease in execution velocity led to an increase in movement complexity, as reflected by higher FuzzyEn values during the slow-velocity (SV) condition, suggesting that moving at deliberately reduced speeds imposes greater movement-control demands.

This finding suggests that both the magnitude and the structure of motor variability are sensitive to execution velocity, albeit in different ways. While SD of the variability reflects the mechanical output of the movement, do not fully capture the adaptive control strategies involved. In contrast, the structure of variability appears more sensitive to movement-control demands and control complexity.

Altogether, the results indicate that motor variability reflects the system’s response to changes in execution velocity. Our findings suggest that slow execution may serve as a strategy to enhance movement-control demands during resistance exercise. However, the practical application of variability measures to guide or assess strength training interventions remains an open question and warrants further exploration in future research. Additionally, it should be explored how prolonged exposure to these velocity conditions affects long-term adaptive capacity, as well as the role of fatigue or other constraints (e.g., contraction type or load) in the evolution of motor complexity and variability.

## Figures and Tables

**Table 1:** Participant Characteristics: Gender, sample size, age, height, body mass, 1RM squat, 1RM/body mass.

Gender	n	Age (Years)	Height (cm)	Body Mass (kg)	1RM Squat (kg)	1RM/Body Mass
**Male**	37	25.9	175.1	73.5	112.9	1.5
**Female**	35	25.4	163.2	61.6	77.1	1.3

## Data Availability

The data that support the findings of this study are available from the corresponding author upon reasonable request. Similarly, the codes used to perform the variability analyses are available upon request to the corresponding author.
